# A novel radiological classification of the distal humerus: morphometric analysis across two European populations

**DOI:** 10.1016/j.xrrt.2026.100790

**Published:** 2026-06-02

**Authors:** Jorge H. Nuñez, Giacomo Traverso, Ainhoa Alvarez Valdivielso, Maria José Jimenez-Jimenez, Laura Rey-Fernández, Thorsten Gehrke, Mustafa Faith Dasci, Mustafa Citak

**Affiliations:** a Department of Traumatology and Orthopedic Surgery, Hospital Universitari MútuaTerrassa, Terrassa, Barcelona, Spain; b Department of Traumatology and Orthopedic Surgery, University Hospital San Martino, Genova, Italy; c Department of Traumatology and Orthopedic Surgery, Hospital Universitari Son Espases, Palma de Mallorca, Spain; d Department of Orthopaedic Surgery, ENDO-Klinik Hamburg, Hamburg, Germany

**Keywords:** Distal humerus, Morphometry, Total elbow arthroplasty, Classification system, Population differences, Radiographic analysis

## Abstract

**Background:**

Total elbow arthroplasty (TEA) utilization is projected to increase substantially, with revision TEA expected to outnumber primary procedures by 2050. Aseptic loosening remains the most common indication for TEA revision. This study aimed to develop and validate a novel morphometric classification system for the distal humerus to optimize pre-operative planning and implant selection.

**Methods:**

A retrospective analysis of 300 anteroposterior elbow radiographs from patients in Spain (n = 138) and Germany (n = 162) was conducted. The morphometric index was calculated as the ratio of medullary canal widths at 120 mm and 35 mm from the lateral epicondyle. Methodology follows the validated percentile-based approach introduced by Citak et al, adapted here to the context of distal humeral morphology. Percentile-based classification thresholds were established: type A (≤25th percentile, ≤0.239), type B (25th-75th percentile, 0.239-0.336), and type C (>75th percentile, >0.336). Interobserver and intraobserver reliability were assessed. Statistical analyses included analysis of variance (ANOVA), *t*-tests, and chi-square tests to evaluate morphometric differences across demographic groups.

**Results:**

The morphometric index ranged from 0.134 to 0.523 (mean 0.29 ± 0.07). Classification yielded type A: 75 cases (25%), type B: 150 cases (50%), and type C: 75 cases (25%). Interobserver reliability was excellent (r = 0.873, *P* < .001), with outstanding intraobserver reproducibility (r > 0.97). Significant geographical differences were observed, with Spanish patients exhibiting higher morphometric indices than German patients (0.317 ± 0.071 vs. 0.266 ± 0.060, t = 6.747, *P* < .001) and greater type C prevalence (38.4% vs. 13.6%, χ^2^ = 31.508, *P* < .001). Females demonstrated significantly higher indices than males (0.322 ± 0.068 vs. 0.268 ± 0.063, t = −7.009, *P* < .001), with type A being male-dominant (86.7%) and type C female-dominant (62.7%). Patients over 65 years showed a higher prevalence of type C morphology (33.3%).

**Conclusion:**

This novel morphometric classification system demonstrates excellent reliability and reveals significant population-based and sex-related variations in distal humeral anatomy. These findings support the clinical relevance of population-specific morphometric considerations in TEA planning and implant design.

Although total elbow arthroplasty (TEA) remains a relatively rare procedure, projections indicate a steady rise in utilization over the next decades, with revision TEA expected to increase nearly 5 times faster than primary TEA and to outnumber it by 2050.^12^^,^^22^ The most common indication for revision is aseptic loosening.^4^^,^^27^ Because revision TEA both worsens patient outcomes and incurs substantially higher costs than primary TEA, identifying modifiable risk factors to reduce revision rates is of critical clinical and economic importance.^22^^,^^23^^,^^28^ In addition to surgical technique and indication, several patient-related factors may contribute to implant failure around the elbow, including age, sex, obesity, smoking, and high comorbidity.^10^^,^^13^^,^^21^ Another key element is the anatomy of the distal humerus, which exhibits marked inter-patient variability.^9^^,^^30^ When planning TEA with stemmed implant components, it is crucial to account for these morphological differences.^9^^,^^16^^,^^30^ A thorough understanding of distal humeral anatomy is therefore essential to optimize surgical planning and guide appropriate implant selection in elbow arthroplasty.^9^^,^^16^^,^^29^

Despite its surgical complexity and critical role in elbow reconstruction, the distal humerus has been comparatively understudied from a morphometric perspective.^9^^,^^16^^,^^29^^,^^30^ This gap is notable when contrasted with the substantial literature on lower-extremity morphometrics, particularly of the distal femur, where sex- and population-related variability has been thoroughly characterized to refine component geometry and sizing for surgical planning and implant selection.^8^^,^^17^ Although demographic variability in humeral anatomy has been explored, prior studies have largely emphasized global or diaphyseal indices for anthropometric or forensic purposes, rather than developing clinically oriented morphometric frameworks for the distal segment.^16^^,^^26^^,^^30^ As a result, surgeons currently lack an evidence-based, reproducible classification to anticipate distal humeral canal geometry and cortical constraints during pre-operative planning.^9^^,^^16^ Such limitations are clinically relevant, as the geometry of the medullary canal—similar to the distal femur—can directly affect implant fit and stability, and may contribute to stem loosening.^16^^,^^25^

Recent radiological classifications, such as the one proposed by Citak et al for the distal femur, have introduced reproducible morphometric frameworks based on standardized ratios derived from radiographs.^3^ This methodology demonstrated that anatomical variations could be systematically categorized using percentile-based cutoffs, creating clinically relevant morphological types that account for population variability while maintaining reproducible classification criteria.^2^^,^^5^^,^^7^ This approach has proven particularly valuable for pre-operative planning and implant selection, offering a more nuanced understanding of anatomical diversity than traditional classification systems.^2^^,^^7^^,^^18^ Building on this rationale, the aim of this study was to develop and validate a novel morphometric classification system for the distal humerus using anteroposterior (AP) radiographs from patients in 2 European populations. By calculating a ratio between medullary canal widths measured at standardized distances from the joint line, we sought to (1) classify patients into distinct anatomical types, (2) assess the interobserver and intraobserver reliability of the measurements, and (3) explore differences in morphometric types by country and sex. We hypothesized that a percentile-based morphometric classification could provide a reproducible framework to describe distal humeral anatomy, while serving as a descriptive and hypothesis-generating tool for future studies investigating its clinical implications.

## Materials and methods

### Study design and population

This retrospective radiological study analyzed AP radiographs of the distal humerus from 300 patients across 2 countries: Spain (n = 101) and Germany (n = 162). This retrospective study was performed after obtaining approval from our institutional review board and our local ethics committee. The study population included 170 males (64.7%) and 92 females (35.3%) with a mean age of 56.2 ± 19.1 years (range: 18-89 years). We excluded from further analysis patients younger than 18 years of age, as well as those with external radiographs, those with elbow arthroplasty and other implants in situ, patients with evidence of elbow pathology, fractures, or degenerative changes that could affect morphometric measurements, and cases with inadequate radiographic quality or positioning that would compromise accurate measurements.

#### Radiological measurements

Two standardized measurements were performed on AP radiographs of the elbow: measurement at 120 mm proximal to the elbow joint line (Med_120 mm) and measurement at 35 mm proximal to the elbow joint line (Med_35 mm). These measurements represented the medullary canal width at the specified distances from the joint line. All measurements were performed digitally using standardized radiological software with consistent magnification correction. For both the German and Spanish cohorts, measurements were independently performed by 2 experienced orthopedic surgeons (observer A and observer B) to assess interobserver reliability. Additionally, both observers repeated their measurements after a 2-week interval to evaluate intraobserver reliability, ensuring consistent measurement techniques throughout the study.

#### Morphometric classification system

**Figure 1:**
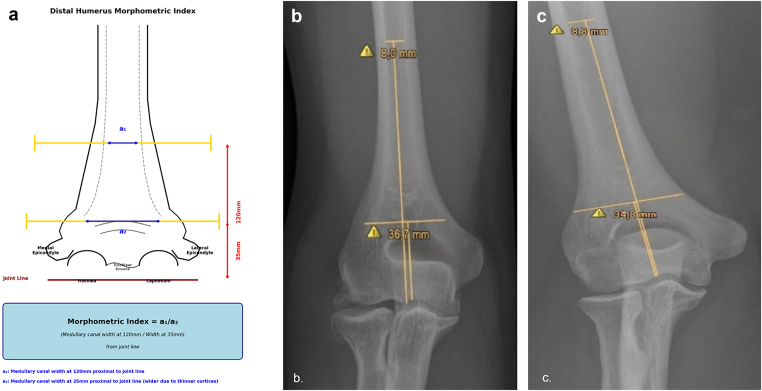
Morphometric index calculation adapted to the distal humerus. (**a**) Schematic representation showing standardized measurements of the medullary canal width at 120 mm (Med_120 mm) and at 35 mm (Med_35 mm) proximal to the elbow joint line. (**b** and **c**) Examples of measurements performed on anteroposterior elbow radiographs, illustrating the practical application of the methodology.

Following the methodology described by Citak et al^3^ for distal femur classification, we developed a ratio-based classification system for the distal humerus. Citak's approach established a morphometric classification based on the principle that anatomical variations in bone geometry can be systematically categorized using ratio indices derived from standardized measurements. Their method utilized percentile-based cutoffs (25th and 75th percentiles) to create 3 distinct morphological types, providing a reproducible framework for classifying bone morphology that accounts for population variability while maintaining clinical relevance.^3^ Adapting this validated methodology to the distal humerus, our morphometric index was calculated as the ratio of the medullary canal width at 120 mm to the width at 35 mm proximal to the joint line: Morphometric Index = Measurement at 120 mm/Measurement at 35 mm (Fig. 1, *a-c*). Based on the distribution of this index ratio across the entire cohort, patients were classified into 3 morphometric types according to the 25th and 75th percentiles: type A (low ratio) corresponding to values ≤ 25th percentile, type B (intermediate ratio) encompassing values between the 25th and 75th percentiles, and type C (high ratio) representing values > 75th percentile.

### Statistical analysis

Statistical analysis was performed using R version 3.3.3 (The R Foundation for Statistical Computing, Vienna, Austria). Descriptive statistics including means, standard deviations, and ranges were calculated for all continuous variables, while frequencies and percentages were reported for categorical variables. Interobserver and intraobserver reliability were assessed using Pearson correlation coefficients with 95% confidence intervals. Differences between groups were analyzed using independent *t*-tests for continuous variables and chi-square tests for categorical variables. The normality of data distribution was assessed using the Shapiro-Wilk test. One-way analysis of variance (ANOVA) was used to compare morphometric indices between the 3 classification types, followed by post hoc *t*-tests with Bonferroni correction for pairwise comparisons. Statistical significance was set at *P* < .05 for all analyses.

## Results

### Morphometric index distribution and classification

**Figure 2:**
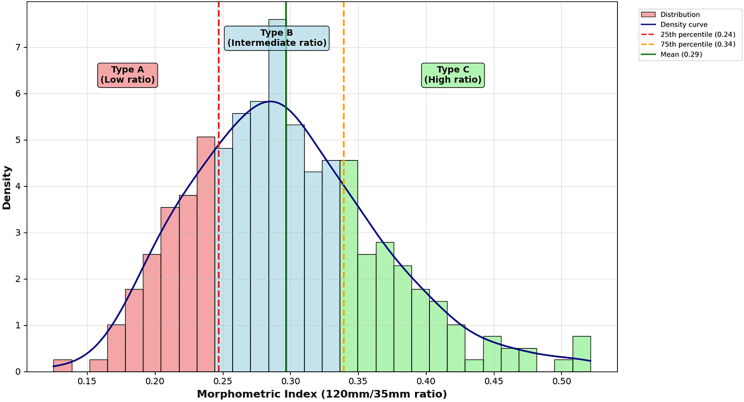
Distribution of morphometric index in the study cohort. The histogram illustrates the spread of index values (range: 0.134-0.523) with classification thresholds marked at the 25th percentile (<0.24) and 75th percentile (>0.34). Based on these cutoffs, 3 morphometric types were defined: type A (<0.24), type B (0.24–0.34), and type C (>0.34).

The morphometric index (120 mm/35 mm ratio) ranged from 0.134 to 0.523 across the entire cohort, with a mean value of 0.29 (standard deviation: 0.07) (Table I). The 25th percentile was 0.239 and the 75th percentile was 0.336, establishing the classification thresholds. The final classification yielded: type A (low ratio ≤0.24): 75 cases (25%), type B (intermediate ratio 0.24-0.34): 150 cases (50%), and type C (high ratio >0.34): 75 cases (25%) (Fig. 2).

**Table I:** Summary of demographic and morphometric characteristics of the study cohort.

N	300
Age (yr) = (median [range])	55.1 [18-99]
Sex = male/female (%)	180/120 (60.2/39.8)
Country = Germany/Spain (%)	162/138 (54.2/45.8)
Side = left/right (%)	100/100 (50.0/50.0)
AP diameter at 12 cm = (median [range])	1.03 [0.41 - 1.73]
AP diameter at 3.5 cm = (median [range])	0.4 [1.84 - 5.50]
Novel Index Ratio = (median [range])	0.29 [0.13 - 0.52]

*AP*, anteroposterior.

### Reliability analysis

Interobserver reliability for the morphometric index demonstrated excellent correlation (r = 0.873, *P* < .001), with a mean difference of −0.038 (standard deviation: 0.034). Intraobserver reliability showed excellent reproducibility for both observers (observer A: r = 0.999, *P* < .001; observer B: r = 0.971, *P* < .001).

#### Morphometric characteristics by classification type

**Figure 3:**
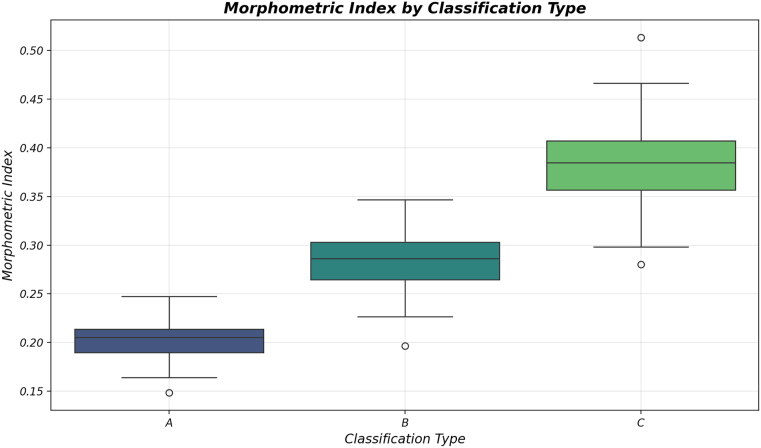
Morphometric index by classification type (box plot).

The 3 morphometric types showed distinct and statistically significant differences (ANOVA F = 623.426, *P* < .001). Post hoc analysis revealed statistically significant differences between all pairwise comparisons (type A vs. B: t = 21.267, *P* < .001; type A vs. C: t = 31.919, *P* < .001; type B vs. C: t = 21.213, *P* < .001) (Fig. 3).

### Geographical, age-, and gender-related differences

**Figure 4:**
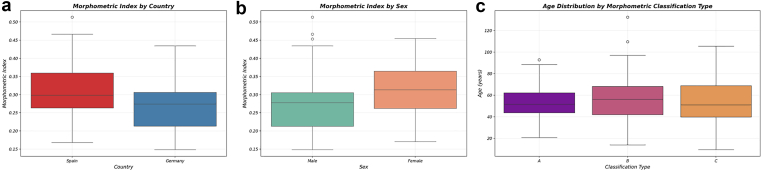
Box plots of the morphometric index. (**a**) Comparison of morphometric index values between countries, showing significantly higher indices in the Spanish cohort. (**b**) Comparison of morphometric index values by sex, with females demonstrating higher indices than males. (**c**) Age distribution across morphometric classification types, illustrating a higher prevalence of type C morphology in older patients.

Comparative analysis revealed significant geographical variation (Table II). Patients from Spain exhibited a significantly higher mean morphometric index than those from Germany (0.304 ± 0.063 vs. 0.266 ± 0.060, t = 4.851, *P* < .001) (Fig. 4, a). Moreover, the distribution of classification types differed markedly between countries (χ^2^ = 19.150, *P* < .001), with a higher prevalence of type C in the Spanish cohort (38.6% vs. 16.7%).

**Table II:** Patient characteristics stratified by morphometric group and sex.

Variable	Male			Female		
	Group A	Group B	Group C	Group A	Group B	Group C
Age (yr) (median [range])	54.00 [20.00-92.00]	55.00 [18.00-93.00]	46.00 [20.00-85.00]	42.00 [23.00-78.00]	55.00 [18.00-97.00]	68.50 [26.00-99.00]
AP diameter at 12 cm (median [range])	0.87 [0.44-1.16]	1.09 [0.81-1.69]	1.24 [0.88-1.56]	0.76 [0.41-0.96]	0.94 [0.72-1.26]	1.15 [0.81-1.73]
AP diameter at 3.5 cm (median [range])	4.09 [2.98-5.50]	3.93 [2.83-5.37]	3.27 [2.44-4.07]	3.26 [2.36-4.08]	3.28 [2.33-4.50]	3.03 [1.84-4.29]
Novel Index Ratio (median [range])	0.217 [0.125-0.247]	0.287 [0.247-0.336]	0.372 [0.340-0.518]	0.224 [0.174-0.235]	0.295 [0.250-0.339]	0.385 [0.340-0.521]

*AP*, anteroposterior.

Sex-based analysis also showed marked differences; females had significantly higher morphometric indices compared to males (0.322 ± 0.068 vs. 0.268 ± 0.063, t = −7.009, *P* < .001) (Fig. 4, b). Type A was predominantly composed of male patients (86.7%), while type C was female-dominant (62.7%). Type B exhibited a more balanced sex distribution (male 58.0%; female 41.3%). These differences were statistically significant (χ^2^ = 39.630, *P* < .001).

Age demonstrated no significant linear correlation with the morphometric index (Pearson r = 0.100, *P* = .084; Spearman ρ = 0.087, *P* = .133), and mean age did not differ significantly across morphometric types (ANOVA *P* = .300) (Fig. 4, c). However, age-group analysis revealed a significant association between age and type distribution (χ^2^ = 15.795, *P* = .003). Patients over 65 years showed a higher prevalence of type C morphology (33.3%).

## Discussion

In this study, we developed and validated a novel percentile-based morphometric classification system for the distal humerus. This classification system aligns with prior work in other anatomical regions—particularly the distal femur—where systematic classification systems have improved templating accuracy, informed implant design, and clarified the influence of sex and population on bony geometry.^2^^,^^7^^,^^18^ Using standardized radiographic ratios, we identified 3 distinct morphometric types with excellent statistical separation and demonstrated excellent interobserver and intraobserver reliability, underscoring the reproducibility of the system. Importantly, we found significant differences between countries, gender, and older people.

Similar to the findings of the present study on the distal humerus, percentile-based morphometric classification systems have consistently demonstrated excellent reproducibility across different anatomical regions. In the original distal femur classification, Citak et al reported a median intraobserver reliability of 0.995 (interquartile range: 0.994-0.997) and an interobserver reliability of intraclass correlation coefficient (ICC) 0.997 (95% confidence interval: 0.996-0.998).^3^ Subsequent studies have confirmed these results.^1^ Akkaya et al found excellent interobserver and intraobserver agreement for distal femur morphology, with 93% average pairwise agreement for the index group and 95.5% for the anatomical classification group.^1^ Similarly, van den Eeden et al^6^ demonstrated high reproducibility for the Citak classification, reporting a mean Cronbach's α of 0.96 (range 0.93-0.98) and 95% intraobserver agreement. Comparable results have been reported for other anatomical sites. De Matteo et al^19^ validated a tibial ratio-based classification, with intraobserver reliability of 0.998 and interobserver ICC of 0.998. Likewise, Sangaletti et al reported high reliability for the proximal humerus, with Cronbach's α values of 0.97-0.98 and ICC 0.96-0.98.^24^ Collectively, these findings emphasize that ratio-based morphometric classifications are highly reproducible and can be applied consistently across institutions, supporting their clinical relevance.

Our study identified significant geographic and demographic variability in distal humeral morphology, including differences related to country, sex, and age. Spanish patients demonstrated significantly higher morphometric indices than German patients, with a notably greater prevalence of type C morphology. These findings suggest that distal humeral anatomy is shaped not only by individual variation but also by broader demographic and population-level influences.^20^^,^^26^^,^^29^ Sex-related differences were also striking in our cohort, with type A overwhelmingly male-dominant and type C showing female predominance. Evidence from other anatomical regions has been mixed.^2^^,^^5^ Sangaletti et al^24^ observed a higher prevalence of type C among females and type A among males, while in the tibia, De Matteo et al^19^ and Budin et al^2^ both found that type A morphology—characterized by a narrow diaphyseal canal—was less common in women. Age, by contrast, showed no significant linear correlation with the morphometric index in our study, and mean age did not differ significantly across morphometric types. Nonetheless, patients over 65 years demonstrated a higher prevalence of type C morphology. Similar findings were reported by Budin et al^2^, who found age differences particularly among women, with older females more frequently classified as type C in both the distal femur (*P* < .001) and proximal tibia (*P* < .001). This observation may be linked to age-related osteoporosis, which has been shown to correlate with morphometric variation. Kartal et al^11^ reported that type C morphology was strongly associated with osteoporosis. Despite these geographic and demographic differences, the literature consistently supports the underlying principle of the Citak classification: patients with a more patent metaphyseal canal and higher index ratios are at increased risk of aseptic loosening.^3^^,^^7^^,^^18^ Our findings further reinforce that marked variability across populations, sexes, and age groups highlights the clinical importance of incorporating morphometric considerations into implant design and surgical planning in these cases for the distal humerus.

Similar to the application of morphometric classification systems in knee arthroplasty with rotating hinge implants, from a surgical perspective, the classification system may aid in anticipating technical challenges related to stemmed implants.^7^^,^^14^^,^^15^^,^^18^ By integrating this classification into pre-operative planning, surgeons may be able to tailor implant choice and fixation strategy according to individual anatomy, thereby improving long-term implant survival and reducing the risk of revision. Levent et al^14^ and Ekhtiari et al^7^ found that distal femoral morphology may play a role in the risk of aseptic loosening following hinged knee arthroplasty and should be considered when deciding implant type and fixation in patients. Also, Martínez-Peñas et al^18^ and Ekhtiari et al^7^ found that there was a significantly higher rate of aseptic loosening in Group C compared to Groups A and B in Citak ratio at distal femur. In the case of distal humerus narrow canal geometries (type A) may predispose to difficulties in broaching and an increased risk of intraoperative fracture, while wider morphologies (type C) could compromise stem fixation and contribute to aseptic loosening, a leading cause of revision elbow arthroplasty. This last concept takes a lot of importance because aseptic loosening is the most common indication for revision in TEA.^4^^,^^10^^,^^21^^,^^27^^,^^28^ However, in TEA—where fixation is almost exclusively cemented—this classification should not guide implant selection but rather identify morphologies that may challenge cemented fixation, particularly in achieving an adequate cement mantle and cortical support.

This study has several limitations that should be acknowledged. First, its retrospective design may introduce selection bias, although strict inclusion and exclusion criteria were applied to minimize confounding factors; also, radiographic measurements demonstrated excellent reproducibility. Second, only 2 European populations were included (Spain and Germany), and the results may not be generalizable to other ethnic or geographic groups. Given that population-specific differences were observed, further validation in diverse cohorts is warranted. Third, the use of 2-dimensional radiographs may not fully capture the 3-dimensional anatomy of the distal humerus, including canal shape, torsion, and cortical distribution, which can influence implant fixation. Additionally, elbow biomechanics involve both coronal and sagittal forces, unlike the predominantly coronal loading in the knee, and factors such as anterior flanges or bone grafting are not accounted for in this classification. Finally, clinical outcomes were not assessed; therefore, although the classification may help predict implant stability and risk of loosening, it should be regarded primarily as a descriptive and hypothesis-generating tool. Its role in guiding surgical decision-making remains preliminary and requires validation through prospective studies correlating morphometric types with clinical outcomes. Despite these limitations, our study provides an important step toward a standardized, evidence-based morphometric classification of the distal humerus by offering a reproducible system that accounts for demographic variability. In the broader context, our findings reinforce the principle that systematic morphometric analysis can uncover clinically relevant anatomical variability, ultimately helping tailor surgical strategies to individual patients.

## Conclusion

In summary, we propose a novel, percentile-based morphometric classification of the distal humerus that demonstrates excellent statistical validity and reliability. The classification identifies 3 distinct anatomical types with clear demographic and geographic associations. These findings provide a reproducible framework that may improve pre-operative planning and guide implant design in elbow arthroplasty. Future studies should validate this system across diverse populations and assess its predictive value for clinical outcomes.

## Disclaimers

Funding: No funding was disclosed by the authors.

Conflicts of interest: The authors, their immediate families, and any research foundations with which they are affiliated have not received any financial payments or other benefits from any commercial entity related to the subject of this article.
